# Alterations in the microenvironment and the effects produced of TRPV5 in osteoporosis

**DOI:** 10.1186/s12967-023-04182-8

**Published:** 2023-05-17

**Authors:** Zhi-heng Luo, Jian-xiong Ma, Wei Zhang, Ai-xian Tian, Shu-wei Gong, Yan Li, Yu-xiao Lai, Xin-long Ma

**Affiliations:** 1 Tianjin Hospital, Tianjin University, Jie Fang Nan Road 406, Tianjin, 300211 People’s Republic of China; 2 Tianjin Key Laboratory of Orthopedic Biomechanics and Medical Engineering, Tianjin Hospital, Tianjin, 300050 People’s Republic of China; 3 Centre for Translational Medicine Research & Development, Shenzhen Institutes of Advanced Technology, Chinese Academy of Sciences, 1068 Xue Yuan Avenue, Shenzhen University Town, Shenzhen, 518055 Guangdong People’s Republic of China

**Keywords:** Osteoporosis, Bone, Tissue Microenvironment, TRPV5

## Abstract

The pathogenesis of osteoporosis involves multiple factors, among which alterations in the bone microenvironment play a crucial role in disrupting normal bone metabolic balance. Transient receptor potential vanilloid 5 (TRPV5), a member of the TRPV family, is an essential determinant of the bone microenvironment, acting at multiple levels to influence its properties. TRPV5 exerts a pivotal influence on bone through the regulation of calcium reabsorption and transportation while also responding to steroid hormones and agonists. Although the metabolic consequences of osteoporosis, such as loss of bone calcium, reduced mineralization capacity, and active osteoclasts, have received significant attention, this review focuses on the changes in the osteoporotic microenvironment and the specific effects of TRPV5 at various levels.

## Introduction

Osteoporosis (OP) is a systemic skeletal disease primarily characterized by reduced bone mineral density (BMD), increased bone fragility, bone pain, and fracture susceptibility [1]. Recognized as a systematic disease by the 1990 consensus conference on osteoporosis development, OP is characterized as low bone mass and bone tissue microstructure deterioration, resulting in increased bone fragility and fracture risk [1]. In 1994, the WHO published diagnostic criteria for osteoporosis [2]. According to a recent study published by Juliet E. Compston et al. in The Lancet, OP incidence continues to rise in women over 55 and men over 65 [3]. The International Osteoporosis Foundation reports that over 200 million people suffer from osteoporosis globally [4, 5]. With the continued global aging trend, the cost of treating and caring for OP and related diseases is increasing worldwide.

In recent years, researchers have increasingly focused on the “micro” aspects of osteoporosis research, exploring changes in the tissue microenvironment and downstream signal cascades [6, 7]. These studies shed light on the interplay between microenvironment and disease, providing a theoretical basis for developing osteoporosis drugs and treatments. For example, research has investigated the extra-bone organ regulation of bone metabolism, the relationship between the hypoxia pathway, and osteoporosis regulation involving osteoblasts, osteoclasts, and osteocytes as well as the discovery of a new bone morphotype cell “osteomorphs” [8].

**Fig.1:**
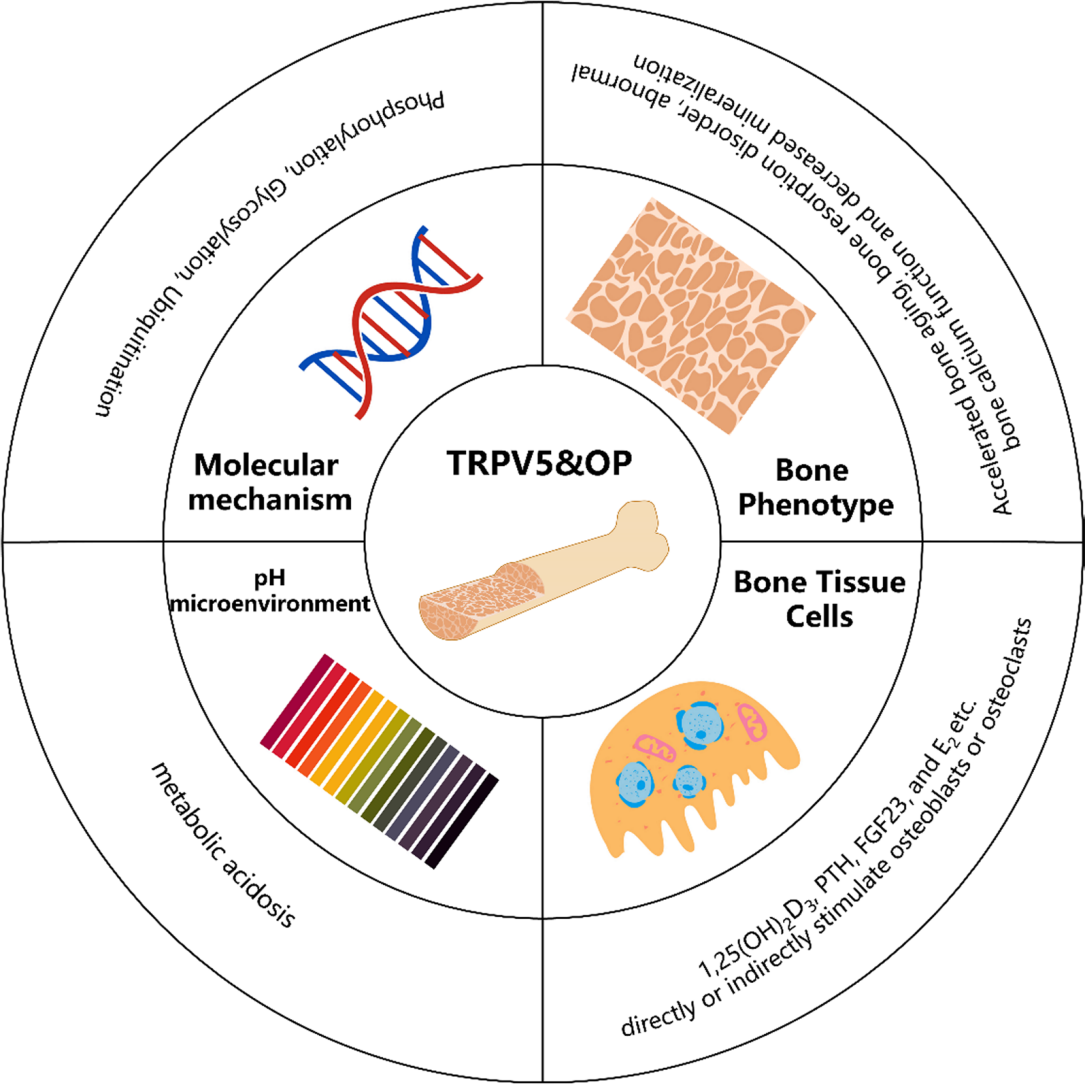
Schematic overview. TRPV5 demonstrates multi-level associations with OP, encompassing such as bone phenotype, bone tissue cells, pH, and molecular mechanism. At the Bone Phenotype level, aberrant TRPV5 function may result in accelerated bone aging, dysregulated bone resorption, and diminished mineralization of bone calcium. The Bone Tissue Cells level primarily involves various hormones acting on TRPV5 to exert effects on distinct bone tissue cells. The Molecular Mechanism level primarily encompasses phosphorylation, glycosylation, and ubiquitination process. Alterations in the pH microenvironment exert a complex impact on the balance of the skeletal system

TRPV5, a member of the transient receptor potential vanilloid (TRPV) family is an ion channel situated on the cell membrane [9]. Investigations concerning the relationship between TRPV5 and osteoporosis (OP) primarily streams from its function in the absorption and translocation of Ca^2+^ icons, as well as its ubiquitous distribution throughout various tissues, including the bone, kidney, and placenta [10–14]. Notably, TRPV5 is distributed in the epithelial cells of both proximal and distal convoluted tubules, playing a pivotal role in modulating urinary calcium [15]. Consequently, it exerts a considerable influence on the systemic regulation of calcium levels. Moreover, the expression and activity of TRPV5 are subject to modulation by diverse microenvironmental factors, such as Ca^2+^, E_2_, 1,25-(OH)_2_D_3_, calcitonin, klotho, PTH, etc. [10–14]. These factors are implicated, either directly or indirectly, in bone metabolism, and their concentrations are influenced by OP. Additionally, TRPV5 is found in osteoclasts, participating in the RANKL signaling pathway and preserving the human bone homeostasis via negative feedback regulation [16]. Furthermore, several researchers have demonstrated, through gene silencing and other methodologies, that TRPV5 represents a promising therapeutic target for patients exhibiting unfavorable prognoses related to bone disorders [17, 18]. Hence, given the inextricable link between TRPV5 and OP, this review delineates the alterations in the microenvironment of OP and explores the underlying mechanisms of TRPV5 at various tissue levels. A comprehensive overview of the association between OP and TRPV5 is illustrated in Fig. 1.

## Microenvironmental characteristics of OP

The onset and progression of osteoporosis can be influenced by a myriad of factors, including age, gender, trauma, and medication [19]. These factors can result in aberrant bone metabolism and subsequent manifestation of osteoporosis. In contrast to health bone tissue, the osteoporosis microenvironment is typified by dysfunctional bone cell activity and anomalous cytokine secretion, culminating in diminished bone mass and skeletal imbalance. This impairs the preservation of normal bone mass and density [20, 21]. Moreover, alterations in the composition of the bone matrix, such as the reduction in collagen fibers and bone mineral salt content, may influence the mechanical properties of the skeletal system [22]. Fluctuations in the pH of the microenvironment can further exacerbate bone loss and progression of osteoporosis [23]. Additionally, inadequate blood supply within the osteoporosis microenvironment can results in bone tissue hypoxia and malnutrition, thereby impacting the growth and function of bone cells [24].

### Bone tissue microenvironment in OP

Examining the bone tissue level, alterations in the osteoporotic bone tissue microenvironment encompass the destruction of bone microarchitecture, reduction in bone mass, and modifications in mechanical stimulation among other changes.

The skeleton, often refereed to as the as the calcium and phosphorus reservoir, is the body’s largest system for calcium and phosphorus deposition. It is composed of bone cells and bone matrix [25]. The growth and metabolism of bone tissue are dynamic processes. Healthy bone tissue undergoes a process of forming bone-like structures, depositing calcium and phosphorus salts, and gradually developing into bone [26]. The structure of bone tissue transitions from woven bone to lamellar bone, ultimately forming compact bone, while the interior of cancellous bone primarily consists of numerous trabeculae [26]. When OP occurs, it leads to abnormalities in bone growth metabolism, resulting in the loss of bone matrix and the disruption of bone microarchitecture. This is mainly manifested in the following aspects [27]: First, trabecular thinning and fracturing: normal cancellous bone consists of tiny trabeculae, while OP causes a reduction, fracturing, or disappearance of these trabeculae, resulting in the loosening of the bone tissue structure. Second, decreased trabecular bone volume: the width and volume of trabeculae directly influence the strength and rigidity of bone tissue, thereby causing a weakened biomechanical load. Lastly, abnormalities in trabecular bone structure: abnormal trabecular structure is also a characteristic of osteoporosis. As the degree of trabecular separation increases and the trabecular connectivity rate decreases, the overall structure of bone tissue becomes abnormal.

From the perspective of bone loss, Type I postmenopausal osteoporosis and Type II senile osteoporosis (disuse osteoporosis) exhibit distinct mechanisms of bone loss [28]. Type I osteoporosis is primarily results from heightened osteoclasts activity due to estrogen deficiency following menopause in women, leading to bone loss that surpasses bone reconstruction, and consequently increasing the risk of bone fractures. One of the main causes of Type II osteoporosis is the skeleton aging, characterized by the accumulation of bone marrow adipose tissue (BMAT) [29].

Bone marrow adipose tissue can adversely impact bone formation, as bone marrow adipose cells and osteoblasts share a common precursor cell, resulting in a negative correlation between them and a decrease in the number of osteoblasts [30]. The abnormal expansion of bone marrow adipose tissue directly influences bone remodelling through the secretion of adipokines and cytokines and exerts a significant detrimental effect on bone homeostasis via negative regulation of hematopoietic mechanisms, exacerbating bone loss [31]. Studies have discovered that the combined effects of MAT accumulation, inflammation, and oxidative stress contribute to the development of osteoporosis [31].

From a biomechanical perspective, the skeleton is a tissue capable of sensing changes in mechanical forces, continuously modulating external mechanical stimuli through the interaction of osteocytes, osteoclasts, and osteoblasts. Guillaume T. Charras et al. found that primary osteoblasts possess a non-selective stretch-activated cation channel with a conductivity of 15pS, and the opening of this cation channel is closely related to the mechanical force stimuli received [32]. In some patients with developmental abnormalities or trauma, changes in their lower limb force lines or lumbar spine sagittal balance parameters may occur. If not corrected in time, long-term abnormal biomechanical stimuli can cause abnormal growth and metabolism of trabecular structures and intraosseous space structures [33].

Additionally, abnormal skeletal force lines are frequently associated with the onset of arthritis, chronic inflammation factors may provoke atypical responses in bone matrix, as well as intraosseous vascular growth and metabolism, leading to abnormal growth of bone joints, diaphysis, epiphyses, and periosteum. Wei Wang et al. showed that patients with lumbar disc herniation may experience lower limb pain, and long-term pain can cause abnormalities in lower limb force lines and lower limb kinematic disorders, leading to muscle and skeletal impairments [34].

Therefore, changes in mechanical force stimulation can affect intraosseous structures, trabecular development, and vascular growth, acting on the bone tissue microenvironment. Insufficient mechanical stimuli have also become one of the pathogenic factors for type II osteoporosis. Furthermore, a recent study by Peng Hui Zhang showed that changes in the biomechanical microenvironment generated by mechanical loading can act on mesenchymal stem cells (MSCs) in three-dimensional scaffolds to promote the expression of osteogenic markers, enhance cell vitality, and inhibit inflammatory factors [35]. As a result, the beneficial effects of mechanical loading on the bone microenvironment can provide new insights for the preparation of bone biomaterials [35]. It is also speculated that this could have a positive impact on chronic inflammatory diseases like osteoporosis (OP) and could serve as a further research direction for OP treatment.

In conclusion, changes in bone microstructure, bone loss, and bone marrow composition, as well as stimulation by the biomechanical microenvironment, can lead to alterations in skeletal growth and development and bone mass accumulation, involving dynamic changes in the bone tissue microenvironment at the tissue level.

### Changes of bone tissue cells in OP microenvironment

Bone tissue primarily comprises osteocytes, osteoblasts, osteoclasts, bone marrow-derived mesenchymal stem cells, and immune cells, among other components [36]. These various cell types interact and regulate bone tissue growth and metabolism. Recently, researchers have aimed to uncover new mechanisms for bone aging and loss by examining the microenvironment from a novel perspective. Studies have demonstrated that, with increasing age, some cells in the bone microenvironment become heterogenous due to senescence, and these cells and their secreted dysfunctional factors are collectively referred to as the senescence-associated secretory phenotype (SASP). SASP plays a role in mediating age-related bone loss [37].

Osteoblasts play a crucial role in bone formation, as they are responsible for the deposition of various bone minerals and type I collagen, and eventually differentiate into osteocytes. Osteocytes primarily serve functions such as mechanical force transmission, regulation of osteoblast activity, control of bone resorption, regulation of PO_4_^3−^ and Ca^2+^ levels, intercellular communication with perivascular cells, remodeling of the surrounding environment, and secretion of relevant hormones, among others [38]. Multiple microenvironmental factors influence the process, including matrix mineralization, extracellular matrix arrangement, oxygen tension, mechanical force, collagen degradation, exogenous molecules, and FGF-2, among others [38]. In the osteoporosis (OP) microenvironment, osteoblasts aging has a significant impact on bone loss. Factors contributing to osteoblast aging include the accumulation of reactive oxygen species (ROS) in the bone microenvironment, DNA damage, and telomere attrition, among others [39]. In the chronic inflammatory microenvironment of OP, aged osteoblasts accumulate and exhibit resistance to clearance by immune cells, subsequently secreting receptor activator of nuclear factor-kappa B ligand (RANKL) [40]. RANKL activates osteoclasts via the RANKL-RANK pathway, exacerbating bone resorption. Consequently, researchers have proposed that eliminating the RANKL secretion in aging osteoblasts and blocking their aberrant interaction with osteoclasts might serve as a new strategy for OP [41]. In this context, an innovative hydrogen peroxide-responsive bone repair complex (HPB@RC)-alendronate (ALN) nanoscale enzyme drug delivery platform was developed to reverse OP progression by scavenging reactive oxygen species (ROS) and silencing the RANKL gene [41]. Additionally, as age advances, osteoblast thickness measurements in the basic multicellular unit (BMU) reveal an inverse relationship with age [42], indicating that the microenvironmental changes induced by osteoblast aging are closely related to bone resorption and can potentiate OP progression by promoting bone loss.

In 1961, the direct external environment of osteocytes was defined as the Grenzscheide or limiting membrane, primarily composed of polysaccharides and extravascular fluid [43]. This membrane serves as a barrier preventing mineralized materials from entering the cavity, thus providing channels for extracellular material transport [43]. Aarden et al. discovered that in vitro, osteocytes can modulate their extracellular biochemical microenvironment by producing osteopontin, osteonectin, osteocalcin, and other molecules [44]. Osteocytes are not only involved in calcium and phosphorus metabolism and endocrine signaling in bone, but also responsible for bone formation due to mechanical stimulation and bone loss induced by disuse [45]. Osteocytes execute complex mechanical sensing between themselves, the environment, and adjacent cells. With their lacunar reticular structure cytoskeleton adhesion, dendrites, intercellular junctions, primary cilia, ion channels, extracellular matrix, and focal adhesion providing a complex microenvironmental system for periosteal cells. As a result, osteocytes can function as a biomechanical sensor to mediate changes in external mechanical stimulation and the intracellular biochemical microenvironment [46]. In patients with disuse osteoporosis, diminished bodily function typically results in slowed cellular metabolism, weakened matrix mineralization capacity, hypoxia, and respiratory acidosis. These changes lead to fluctuations in the body's environmental pH. The lack of osteocyte mechanical stimulation in elderly patients, combined with the aforementioned factors, can engender osteocyte differentiation disorders. Knothe Tate et al. observed in their study of bone histology in osteoporotic patients that the structural integrity of the bone cell network and tissue three-dimensional architecture are altered, resulting in fractures that are prone to occur and difficult to heal due to the imbalance between bone reconstruction and the load capacity [47].

Nelson G et al. reported that senescent cells secrete found that aging cells secrete chemokines, inflammatory factors, and extracellular matrix proteins that generate a toxic microenvironment, affecting neighboring cells and facilitating the accumulation of aging cells and the development of tissue dysfunction [48]. Extracellular vesicles (EVs), including exosomes, microcapsules, and apoptotic bodies [49], that can deliver specific proteins, such as tenascin C, sema4D, microRNA-214-3p, and bone morphogenetic protein 1–7 [50]. Osteoclasts also release EVs to self-regulate, primarily by secreting EVs containing RANK, which competitively inhibits the interaction between the RANK receptor and RANKL on the surface of osteoclasts [51]. Exosomes derived from bone marrow mesenchymal stem cells (BMSCs) can promote bone healing by delivering miRNA [52]. Therefore, alternations in the extracellular microenvironment caused by EVs warrant consideration in developing novel OP therapeutic strategies. Additionally, the interaction between BMSCs and hematopoietic stem cells (HSCs) can also influence the bone marrow cavity microenvironment [53]. SusanK. Nilsson et al. found that megakaryocytes secrete various cytokines affecting the growth and proliferation of HSCs and other hematopoietic cells, which in turn affect bone formation [54]. Recent studies by Chang Jun Li et al. revealed that during aging, senescent immune cells accumulate in the bone marrow and secrete grancalcin protein, which binds to the plexin-B2 (Plxnb2) receptor of BMSCs, inhibiting osteogenesis and promoting adipogenesis [55]. Consequently, grancalcin protein may serve as potential target for the treating age-related osteoporosis [55].

Immune cells play a crucial role in the bone tissue microenvironment [56–58]. Studies have shown that resident macrophages are present in all tissues, with exception of hyaline cartilage [59] and are intimately involved in tissue repair, debris removal, and maintaining the microenvironment homeostasis [60]. Macrophage polarization can enhance osteoblast differentiation, increase osteogenic effects, and facilitate mineralization [61]. The polarization state is also related to the immunosuppressive phenotype generated by the combination of interleukin (IL)-4, IL-10, and transforming growth factor-beta (TGF-β) in the bone microenvironment, which exerts the strongest immunosuppressive effect on M2 macrophage polarization [62]. Studies have shown that the macrophages phenotype (M1) transitions to M2 following IL-4 stimulation when co-cultured with MC3T3 cells [63]. The degree of osteoblast differentiation and osteogenic ability of MC3T3 cells was higher than that of M0 cells co-cultured with MC3T3 cells [63]. Therefore, the in vivo the transformation from M1 to M2 phenotype is essential for tissue growth, healing, osteogenic effects, and osteoblasts function [63]. Joseph Muñoz posits that modulating the fluctuations of various cytokines in the local in vivo microenvironment could treat osteoporosis based on macrophage polarization, representing a novel approach [61]. XU's research indicates that the interaction among monocytes, macrophages, osteoclasts, bone marrow stromal cells, and osteoblasts plays a vital role in the pathological study of OP [64].

Recently, new technologies have emerged for studying bone tissue microenvironment at the cellular level, with single-cell sequencing becoming a popular method for investigating bone tissue metabolic diseases. By extracting and sequencing from disease-affected areas, distinct heterogeneous cell subsets can be selected and compared with public data sets to identify interactions among various cell subsets during bone tissue diseases process. Associating different cell subsets with clinical indicators can help define the pathological state of related bone samples. The CyTOF (Cytometry by Time-Of-Flight) Mass Cytometry method enables high throughput, multi-omics single cell analysis, providing a technical means for detecting cellular changes and alteration in the surrounding microenvironment. Through microenvironment state analysis, sensitive cells exhibiting differential drug effects can be effectively screened, offering support for the identification of potential drug targets.

### Molecular mechanisms of the microenvironment in OP

In this study, we discuss the relationship between the TRPV family of ion channels and OP. Classical signaling pathways in OP, including Wnt/β-catenin, RANK/RANKL/OPG, TGF-β, PI3K/Akt, and Notch, have been widely studied and documented [65].

The TRPV ion channels, a subfamily of transient receptor potential vanilloid (TRPV) receptors, are ubiquitously distributed calcium ion receptor proteins present on the cell membranes of various tissues and organs in living organisms [66]. Based on homology, they can be further classified into TRPV1, TRPV2, TRPV3, TRPV4, TRPV5, and TRPV6 [66]. These proteins comprise six transmembrane domains, with the fifth and sixth domains jointly forming a non-selective cation channel. The N-termini and C-termini of these proteins are located within the cytoplasm, and they exhibit permeability to Na^+^, K^+^, and Ca^2+^ ions [66]. However, different subtypes can respond to various external stimuli, such as pH, temperature, pressure, and osmotic pressure.

TRPV1, commonly referred to as the capsaicin receptor, exhibits sensitivity to Ca^2+^, pH, and chemical stimuli, predominantly inducing downstream pain responses. It has been demonstrated that pain associated with bone loss is intimately linked to TRPV1 [67]. TRPV2 primarily responds to temperature stimuli exceeding 53 °C and contributes to osteoclast differentiation and mediation of Ca^2+^ oscillations governing bone metabolism [68, 69]. TRPV3, denoted as thermo-activated channel, is predominantly sensitive to mild temperature stimuli (usually 30–33 °C) and exhibits the highest expression in keratinocyte-forming cells of the skin [70]. Further investigation is required to elucidate its impact on the osteogenic differentiation of bone marrow stromal cells (BMSCs) [17]. TRPV4 demonstrates heightened sensitivity to mechanical stimuli and is highly expressed in chondrocytes, playing a pivotal role in the proper development of bone growth plates [71]. Research on TRPV5, initiated in 1999 [72], has revealed its exceptional sensitivity and selectivity towards Ca^2+^ ions, as well as its high sensitivity to extracellular pH, which directly influences its activity. Primarily distributed in bone tissue and renal cells, TRPV5 is crucial for regulating Ca^2+^ absorption within the body [73, 74]. TRPV6, another Ca^2+^sensitive ion channel is strictly regulated by Ca^2+^ and 1,25-(OH)_2_D_3_ [14]. A summary of the functions and distributions of various TRPV family ion channels is presented in Table 1.

**Table 1:** TRPV family ion channel function and distribution

Name	Main function	Sensitive materials	Location	References
TRPV1	Regulation: Temperature; Pressure; Osmotic pressure; pH	Capsaicin; Heat; Na^+^; Ca^2+^; Acidosis; H^+^; Endogenous agonists	Variety of tissues and cell types	[75, 76]
TRPV2	Regulation: Temperature Cell migration; Innate immune system	Ca^2+^; Heat	Skeletal muscle; Cardiac muscle; Neurons; Heart; Gastrointestinal tract; Lung; Pancreas; Retina; Brain	[77–85]
TRPV3	Regulation: Temperature; Synaptic plasticity	Ca^2+^; Bradykinin; Histamine; ATP; PKC; PGE_2_; a-hydroxyl acids; 2-APB; PIP_2_; Heat	Tongue; Testis; Skin keratinocytes	[86–96]
TRPV4	Regulation: Mechanical; Osmotic pressure	Ca^2+^; Heat	Chondrocyte; Adipocytes; Vascular endothelium; Retina; Neurons	[97–103]
TRPV5	Regulation: pH; Ca^2+^selective TRP channel; Inward rectification of the current- voltage	Ca^2+^; E_2_; 1,25-(OH)_2_D_3_; Calcitonin; Klotho; PTH; Testosterone; Vasopressin; MUC1; PIP_2_	Bone; Kidney; Placenta	[10–14, 104, 105]
TRPV6	Regulation: Ca^2+^selective TRP channel	Ca^2+^; 1,25-(OH)_2_D_3_; Ba^2+^; Sr^2+^; Mn^2+^; Zn^2+^; Cd^2+^; La^3+^; Gd^3+^; PIP_2_	Intestinal enterocytes; Kidney; Placenta; Uterus; Pancreas; Epididymal epithelium; Brain; Stomach	[14, 106–108]

## Overview of TRPV5

**Fig. 2:**
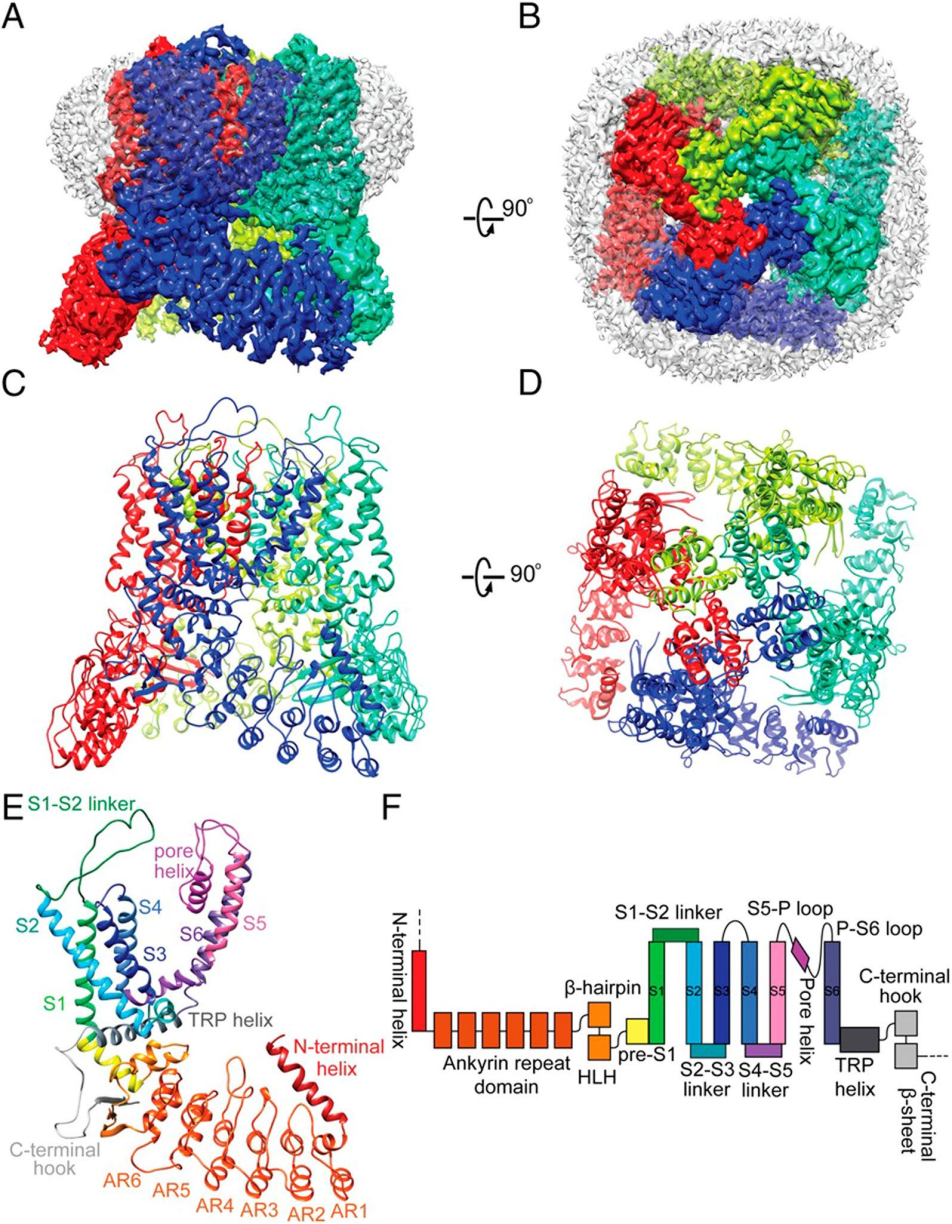
Structural and domain organization of TRPV5 as visualized by cryo-electron microscopy [112]. **A** Lateral perspective of the TRPV5 structure. **B** Overhead perspective of the TRPV5 structure. **C** Lateral representation of the TRPV5 tetrameric assembly. **D** Overhead representation of the TRPV5 tetrameric complex. **E** Lateral depiction of an individual TRPV5 monomeric unit. **F** Schematic illustration of TRPV5 domain organization as originally published by PNAS and authorized for utilization

**Fig. 3:**
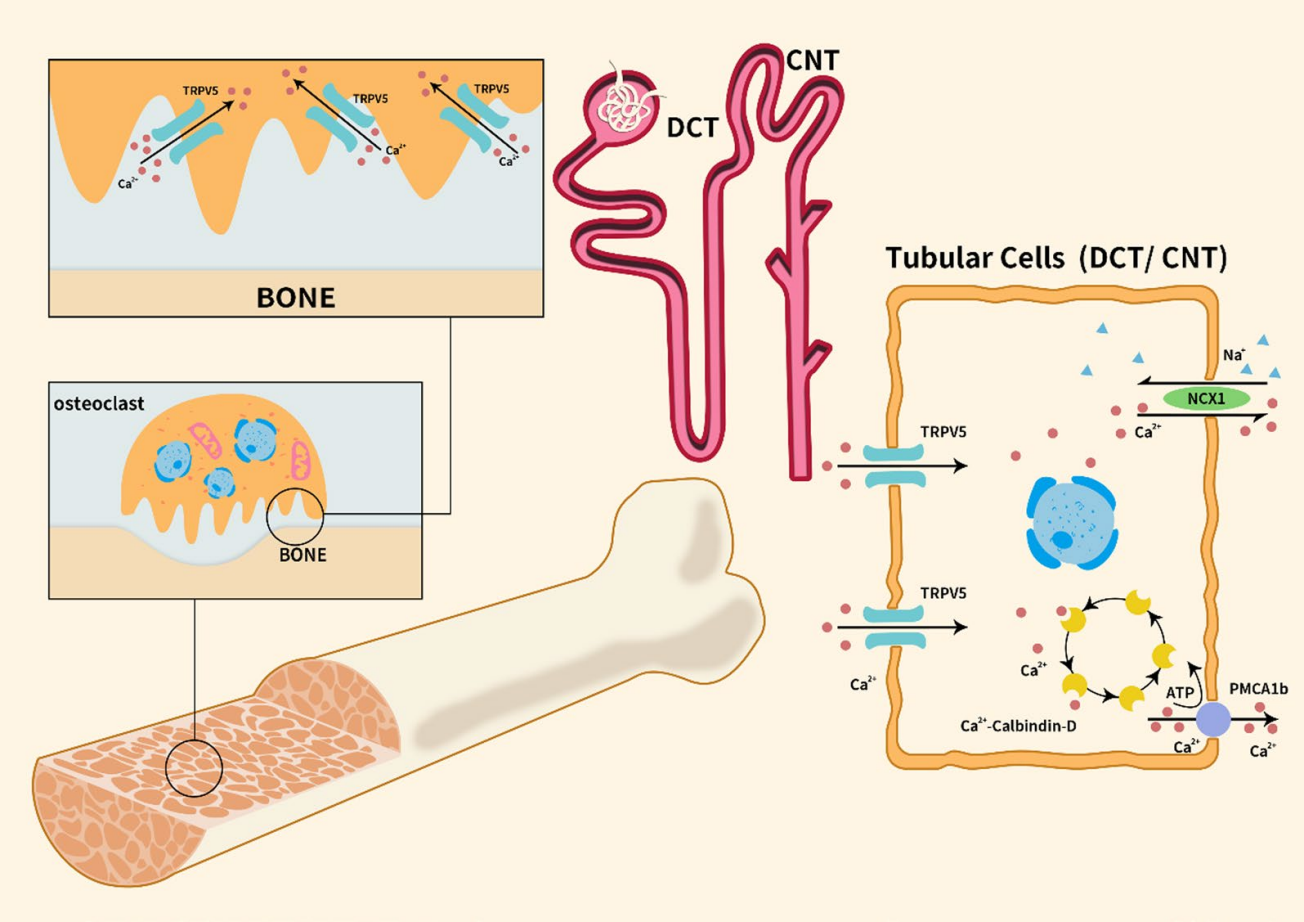
TRPV5 channels are distributed in osteoclasts and the apical membrane of tubular cells in the DCT and CNT. Osteoclasts are typically located in the fossa depression and primarily undergo osteolysis and phagocytosis. The TRPV5 channel is commonly distributed at the folded edge of osteoclasts and mainly performs the transport function of Ca^2+^. The renal tubules are primarily responsible for the reabsorption of Ca^2+^. TRPV5 channels exist on the apical membrane of tubular cells to transport Ca^2+^. When Ca^2+^ enters the cells, it binds with calbindin and is subsequently transported to PMCA1b or NCX1 and pumped out to the other side of the membrane. When the TRPV5 gene is deleted, the reabsorption of renal calcium is significantly reduced, and the excretion of calcium in the urine markedly increased, resulting in the disturbance of calcium levels in the body

As discussed earlier, the primary role of TRPV5 involves mediating Ca^2+^ uptake and transport. Research has demonstrated the indispensability of TRPV5 in epithelial calcium uptake, bone formation, and the regulation of calcium homeostasis, particularly in renal calcium reabsorption, with particular emphasis on renal calcium reabsorption and its intimate connection to bone homeostasis [15, 109, 110]. Notably, TRPV5 functions differently from TRPV1-4, as it is not activated by ligands and is unrelated to the thermosensitive channel family [111]. Dang et al. investigated the three-dimensional protein structure of TRPV5 using cryo-electron microscopy cryo-EM technology [112]; as illustrated in Fig. 2. They discovered that TRPV5's unique characteristics lies in its capacity to modulate the channel's open and closed states via calmodulin binding, a process termed calcium-dependent channel inactivation [112]. Analysis of the W583A structural site revealed an open conformation an open conformation of TRPV5's lower gate, which is highly conducive to calcium hydrate conductivity [112]. Additionally, the group examined the structure of TRPV5-CaM (calmodulin), uncovering two calcium-binding sites within CaM [112]. Calcium influx, ensuing from channel opening, interacts with CaM to close the channel, thereby regulating TRPV5 [112]. Simultaneously, TRPV5 encompasses two CaM-binding sites, with the second C-lobe situated near the lower gate. This arrangement facilitates more efficient effective calcium concentration-dependent channel inhibition [112]. Moreover, the phospholipid phosphatidylinositol 4,5-bisphosphate (PI(4,5)P_2_) functions as an endogenous regulator of TRPV5. Moiseenkova-Bell et al. demonstrated that PI(4,5)P_2_ activates TRPV5 through the direct binding of the N-linker, S4–S5 linker, and S6 helix to TRPV5, enabling Ca^2+^ flow [113]. TRPV5's specific structure dictates its sensitivity to Ca^2+^ [114]. In the renal collecting tubules and distal convoluted tubules, calcium present in the urine enters cells following the concentration gradient via TRPV5, which subsequently transports calcium to the bloodstream through calbindin. Research suggests that this reabsorption mechanism accounts for only 15% of overall renal reabsorption, nevertheless, it holds vital importance in maintaining calcium equilibrium within the body, as illustrated in Fig. 3.

### The relationship between TRPV5 and OP

In recent years, an increasing number of researchers have focused on investigating the association between TRPV5 function and various tissue layers in osteoporosis. The objective of these investigations is to identify the functional targets of TRPV5 by examining its structure, function, and downstream signaling cascade. The outcomes of this research can offer valuable insights and guidance for the development of novel osteoporosis therapies, as well as for enhancing drug efficacy and absorption. Hoenderop JG have suggested that alterations in TRPV5 might be linked to age-related bone disorders, including osteoporosis [10], the relationship is depicted in Fig. 1.

#### TRPV5 and bone phenotype

Eerden et al. reported that TRPV5 plays a significant role in bone resorption and morphological changes [18]. Utilizing gene knockout techniques, they created TRPV5^−/−^ mice and discovered that these mice exhibited disordered bone resorption and decreased femur thickness compared to wild-type TRPV5^+/+^ mice [18]. The absence of TRPV5 led to abnormal osteoclast activity and severe bone resorption disorder in TRPV5^−/−^ mice [115]. Although the number of osteoclasts increased and bone thickness decreased, the mice did not exhibit the phenotype of osteosclerosis, potentially due to partial compensation by the TRPV6 channel [115, 116]. TRPV5 deficiency also impacted bone resorption homeostasis, calcium deposition, and related bone metabolic factors. In male TRPV5^−/−^ mice, the number and volume of trabeculae decreased, the connectivity rate was suboptimal, and the thickness and volume of cortical bone diminished, with a significantly higher internal porosity compared to TRPV5^+/+^ mice [117]. Quantitative backscattered electron (QBEI) measurements of the tibia revealed that CaMean, CaPeak, and CaWidth were positively correlated with age, while CaLow was negatively correlated, and CaMean and CaPeak were lower than in TRPV5^+/+^ mice in young and middle-aged groups [117]. Urinary deoxypyridinoline (DPD) observations indicated that the DPD content of TRPV5^−/−^ mice was related to and proportional to age, and the levels of young and middle-aged TRPV5^−/−^ mice were significantly lower than those of the TRPV5^+/+^ group, suggesting an abnormal bone resorption rate [117]. The lack of TRPV5 accelerated bone aging and disrupted bone resorption. Due to decreased abnormal mineralization of bone calcium function, the cortical bone lumen volume increased, potentially as a result of compensatory mineralization effects [118].

#### TRPV5 and cells of bone and cartilage

TRPV5 is expressed in various cells of bone tissue, including the bone matrix, osteoclasts, and chondrocytes [12]. Osteoclasts release growth factors through bone resorption, which act on chondrocytes to regulate their metabolism and degeneration, as illustrated in Fig. 3 [119]. Several factors can influence the activity of osteoclasts through TRPV5. For instance, 1,25(OH)_2_D_3_, a conventional drug for osteoporosis treatment, can increase Ca^2+^ absorption but also inhibit TRPV5 expression in osteoclasts, leading to the inhibition of early osteoclast differentiation, activity, and bone loss [120]. TRPV5 is also essential for calcium transport in the resorption cavity of osteoclasts. Inhibition of TRPV5 expression by econazole in osteoclasts affected the transport of Ca^2+^ and inhibited bone resorption [121]. In vitro culture of TRPV5^−/−^ mouse osteoclasts showed that the number of osteoclasts was approximately twice that of wild-type (WT) mice, and the number and size of osteoclast nuclei were significantly higher in TRPV5^−/−^ mice [18]. However, the absorptive capacity of osteoclasts was severely impaired. The absence of TRPV5 can increase the number and volume of osteoclasts, but this does not proportionally correspond to the osteoclast effect [18]. Fangjing Chen et al. found that estradiol (E_2_) can increase TRPV5 expression in osteoclasts to inhibit osteoclast differentiation [122]. E_2_ may induce continuous Ca^2+^ oscillations by increasing TRPV5 expression. E_2_ exerts its biological activity mainly by binding with the estrogen receptor ER. Tianwen Ye et al. demonstrated that E_2_ can promote TRPV5 expression and stimulate osteoclast apoptosis by interacting with NF-κB when binding with ER [123]. NF-κB can directly bind to the promoter region of the − 286 nt  to − 277 nt fragment of TRPV5 and promote TRPV5 transcription [123].

Autophagy in chondrocytes is a self-protective mechanism and metabolic mode of cartilage tissue. TRPV5 is expressed in chondrocytes and upregulated during the progression of osteoarthritis. Ismail M. Hdud et al. used immunohistochemistry to identify TRPV5 distribution primarily in the superficial and middle chondrocytes [124]. In a monosodium iodoacetate (MIA) rat model of osteoarthritis, Lunhao Bai et al. observed a positive correlation between the expression of Ca^2+^-dependent proteins, calmodulin, and TRPV5 in chondrocytes of osteoarthritis rats, substantiating the hypothesis that TRPV5 is a calmodulin-dependent channel [113]. The primary mechanism entails the upregulation of TRPV5 expression, which facilitates Ca^2+^ influx, resulting in calcium overload and subsequent activation of calmodulin and CaMKII proteins. CaMKII proteins phosphorylate Beclin1, inhibiting autophagosome formation and consequently suppressing chondrocyte autophagy, which is also stimulated by associated inflammatory factors [113]. Moreover, a subsequent investigation revealed that TRPV5 expression influences chondrocyte apoptosis [125]. The upregulation of TRPV5 promoted chondrocyte apoptosis by augmenting the expression of several apoptosis-related proteins, including calmodulin, DAP, Cleaved caspase-3, Cleaved caspase-6, Cleaved caspase-7, and Cleaved caspase-8 [125].

The presence of TRPV5 in osteoblasts is a matter of ongoing debate among researchers. Bram C. J. van der Eerden and Lieben L believe that TRPV5 is not expressed in osteoblasts, thus supporting the notion of absent of TRPV5 expression [15, 18]. In contrast, Li et al. argue that the complete absence of TRPV5 in osteoblasts cannot be unequivocally confirmed, as although direct detection of TRPV5 was not achieved, they observed downstream calcium-binding proteins and mRNA of calcium transporters. This finding implies that the expression of TRPV5 in osteoblasts may necessitate specific activation conditions [126].

#### TRPV5 and pH in OP

The relationship between in vivo pH and osteoporosis has been a topic of interest among researchers for decades. In 1968, Pellegrino et al. suggested that inorganic salts in the bone system could function as a buffer matrix, and osteoporosis was thought to result from the absolute loss of bone minerals, which played a buffering role in absorbing acidic substances when the bone was “dissolved” [127]. However, pathological states such as uremia, renal acidosis, chronic obstructive pulmonary disease (COPD), and lung transplantation can exert complex effects on the skeletal system. In COPD patients, decreased respiratory function often leads to an elevation in inflammatory factors, which can alter the signaling pathway of bone tissue cells, further enhancing osteoclasts activity and disrupting the balance of bone metabolism, culminating in osteoporosis. Pulmonary dysfunction in COPD patients is frequently accompanied by chronic respiratory acidosis, which increases the amount of H_2_CO_3_ in the body and elevates the H^+^ concentration, causing a decline in tissue pH and resulting in an acidic microenvironment. In untreated or severe COPD patients, hypercapnia and hypoxia can significantly impact bone metabolism, making patients more susceptible to developing osteoporosis [128, 129].Previous investigations have demonstrated that the acidic microenvironment can induce an increase in osteoclast activity, trigger changes in downstream cell signalling pathways, inhibit osteoblast proliferation, and reduce osteoblast mineralization [130].Similarly, in patients with chronic renal insufficiency, there is a decrease in renal metabolic function, resulting in a decline in plasma HCO_3_^−^ and tissue pH, leading to acidosis. Consequently, phosphate and carbonate in bone tissue are lost as buffer substances, and renal tubular epithelial cells exhibit reduced calcium reabsorption and increased urinary calcium excretion, which can contribute to osteoporosis [131]. A study revealed that elevated urinary levels of Ca^2+^ and Mg^2+^ in mice with metabolic acidosis led to a decrease in the gene and protein abundance of TRPV5 and Calbindin-D_28K_ in the kidney [132]. However, effective inhibition of active Ca^2+^ reabsorption was observed in TRPV5^−/−^ mice, indicating that metabolic acidosis did not affect urinary calcium excretion in mice. Additionally, Mg^2+^ exerted an inhibitory effect on TRPV5 activity [105]. Thus, it can be inferred that metabolic acidosis plays a critical role in urinary calcium excretion by mediating the expression abundance of TRPV5.

In recent years, numerous pH-responsive drugs have been developed to target local pH changes in osteoporotic tissues. Dou et al. reported the development of cerium bioactive nanoparticles with pH response, which can target the acidic extracellular microenvironment and inhibit the activity of mature osteoclasts (mOCs). During bone remodeling, the pH value of the bone resorption cavity can reach 3–4, with mOCs triggering the extracellular acidic microenvironment through ATPase H^+^ Transporting V0 Subunit D2 (ATP6v0d2) [133]. Yi Hu et al. argue that in the acidic microenvironment of OP, measures to neutralize acidity are unable to effectively inhibit osteoclasts [134]. Mature osteoclasts can secrete large amounts of H^+^ into the bone resorption area. Based on the unique acidic microenvironment surrounding osteoclasts, the team designed an osteoclast microenvironment-responsive nanoplatform, HA-MC/CaCO_3_/ZOL@PBAE-SA (HMCZP). PBAE, a pH-responsive polymer, can release ZOL in the acidic microenvironment of OP, thereby inhibiting osteoclast activity. Thus, several studied have confirmed the extracellular acidic microenvironment caused by osteoporosis. This acidic microenvironment simultaneously inhibits the expression of TRPV5 ion channels. The exact mechanism by which TRPV5 is affected by the acid–base environment is not entirely clear, but studies have reported the involvement of an acid-sensitive receptor on the basal side of cells in the proximal tubule of the kidney (PT) [9]. When the pH decreases, basal acid-sensitive receptor GPCRs are triggered, leading to phosphorylation of the intracellular pH sensor Pyk2 and activation of the apical Na^+^–H^+^ exchanger type 3 in PT [9]. Yeh et al. found that two extracellular loops in the fifth and sixth transmembrane domains of TRPV5 may form an open structure that plays a major role in the binding and regulation of external H^+^ [73]. Additionally, the mutation from glutamic acid 522 to glutamine E522Q in TRPV5 can reduce the sensitivity of cells to external acidification and alter channel activity [73]. Therefore, it is hypothesized that glutamic acid 522 may act as a pH sensor, and external H^+^ influences the biological activity of TRPV5 by altering the conformation of TRPV5 [73]. Moreover, vesicular transport has been reported, which is termed “kiss and linger” [135]. When the extracellular environment is continuously alkalized, vesicles containing TRPV5 are rapidly recruited to the surface of the cell membrane without collapsing into the plasma membrane. The activity of these vesicles containing functional TRPV5 increases with increasing pH [135]. Conversely, when the extracellular pH value continuously decreases, vesicles containing TRPV5 are withdrawn from the plasma membrane and reach the extracellular space through the transient opening of vesicles, inhibiting the biological activity of TRPV5 [135]. Furthermore, other pathways for the inhibition of TRPV5 activity under acidic conditions have been reported. Edwin C. Fluck et al. [110] found that the activation of PI (4,5) P_2_ was prevented under low pH conditions to suppress TRPV5 activity, revealing a synergistic effect of pH and lipid co-factor in gating the channel. The transition of TRPV5 channel from open to closed conformation under low pH environment was captured using cryo-electron microscopy. These structural and molecular insights provide new understanding of the interplay between pH and TRPV5 gating.

The multifaceted mechanisms through which H^+^ modulates TRPV5 ion channel activity in bone cells in response to alterations in extracellular pH may offer promising opportunities for the innovation of drug delivery approaches and the identification of novel therapeutic targets for osteoporosis treatments.

#### Molecular mechanism of TRPV5 in OP

Calcium absorption in the body relies on the distal tubules of the small intestine and kidney. TRPV5 serves as the primary Ca^2+^ gated channel, and its functionality is contingent on several proteins, including CaR, CaM, 80 k-H, S100A10-annexin II, and calbindin 28 K [11]. CaR is an extracellular Ca^2+^ receptor that exhibits high sensitivity to plasma Ca^2+^ concentrations. Catalin N. Topala et al. discovered that CaR is predominantly located in the distal convoluted tubules (DCT) and connecting tubules (CNT) of the kidney [136]. Cell transfection experiments using HEK293 cells revealed that co-expression of CaR and TRPV5 notably increased intracellular Ca^2+^ concentrations. Patch clamp techniques demonstrated that CaR could stimulate the enhancement of TRPV5 activity (Fig. 3) [136]. 80 k-H is a substrate of PCK. The interaction between 80 k-H and TRPV5 can inhibit the Ca^2+^ influx, increase the TRPV5 sensitivity to Ca^2+^, and expedite channel feedback inhibition [11]. S100A10-annexin II is an essential protein in plasma membrane transport and insertion, exerting its influence through TRPV5 binding [11]. One study indicated that the membrane protein PIRT (phosphoinositide-interacting regulator of TRP) contains a cholesterol-recognition amino acid consensus (CRAC), which can play a significant role in the mechanical regulation of TRP ion channels by binding with cholecalciferol and oxytocin [137]. Although the direct regulations of TRPV5 and TRPV6 remains unclear, it is hypothesized that PIRT regulates TRPV5 through CaM binding [137]. Recently, new functional interactions between TRPV5 and CaM have been reported, characterizing dynamic lobe-specific CaM regulation and persistent interaction with apo-CaM. These two novel interactions play critical roles in rapid inhibition and conformational modulation of the TRPV5 channel, offering new insights into Ca^2+^ transport in the kidney [138].

Protein post-translational modification (PTM) is a crucial mechanism that enables proteins to function [139]. Covalent reactions occur during or after translation, and various chemical groups can be covalently linked with proteins or amino acids, such as methyl, acetyl, phosphate group, sugar chain, and ubiquitin groups. This process allows for the expression, activity, and function of proteins [140, 141]. In TRPV5, the most common modifications include phosphorylation, glycosylation, ubiquitination, and others [142].

1. When the extracellular calcium concentration is low, the release of PTH is promoted, and TRPV5 becomes more sensitive to PTH [143]. TRPV5 has a PKA phosphorylation site, and the cAMP-PKA pathway activates TRPV5 to promote the influx of Ca^2+^ [143]. The catalytic subunit of PKA can directly increase the channel open rate by phosphorylating TRPV5 [144]. By co-expressing PTH and TRPV5 in HEK293 cells, it was found that PTH can stimulate the rapid phosphorylation of threonine-709 on TRPV5, increase the channel open rate, and enhance the Ca^2+^ influx [144]. Additionally, TRPV5 activity stimulation by PTH can occur through the PLC-PKC pathway, and PKC pathway activation can weaken the endocytosis of TRPV5 vesicle transport and increase channel activity [145]. Deletion of two phosphorylation sites in TRPV5 prevents it from responding to PTH stimulation [146]. Histidine phosphorylation is also a PTM, reported by the Xinjiang Cai team, which found that the histidine kinases NDPK-B and PTH1 in mammals can directly regulate the activity of the TRPV5 channel on the plasma membrane by reversible histidine phosphorylation [146].
2. N-glycosylation is a critical process in TRPV5 regulating channel function. There is an N-glycosylation site on asparagine N-358 between 1–2 transmembrane segments. Klotho is a β-glucuronidase that promotes channel activity by hydrolyzing N-glycan residues on TRPV5 [74]. If β-glucuronidase is used to interfere with cells with mutations in the N-358Q site, the TRPV5 channel exhibits no stimulation response, while HEK293 cell TRPV5 can produce a Ca^2+^ influx response to stimulation [74]. Additionally, this study demonstrates that vitamin D can upregulate Klotho and TRPV5 to enhance the kidney's absorption of urinary calcium and reduce urinary calcium excretion to maintain normal blood calcium levels [74]. Jinho Lee et al. found that soluble Klotho can anchor TRPV5 on the membrane surface by combining with TRPV5 and the membrane protein Galectin-1, upregulating TRPV5 without FGF23 while preventing the degradation of TRPV5 and endocytosis caused by diabetes [147].A recent report by Miguel Chillón’s team demonstrated that the anti-aging gene α-Klotho produces two major transcript variants, processed Klotho (p-KL) and secreted protein (s-KL), which exert distinct effects on mineral metabolism and bone microstructure [148]. Treatment with p-KL was found to upregulate TRPV5 expression but had detrimental effects on calcium and phosphate ion metabolism and bone homeostasis. In contrast, s-KL did not exhibit these effects and instead had beneficial effects on bone mass and microstructure, suggesting its potential as a long-term therapeutic molecule for age-related defects.
3. Ubiquitination is a process that requires the coordinated participation of ubiquitin activator (E1), ubiquitin binding enzyme (E2), and ubiquitin ligase (E3). These enzymes interact with the target protein, specifically modifying it, and thereby regulating protein function [143]. Tasaki T et al. identified UBR4/P600 as a prominent characteristic of ubiquitin E3 ligase in TRPV5 through mass spectrometric analysis of coimmunoprecipitation [149]. UBR4 serves as TRPV5's primary binding partner, recognizing and interacting with proteins containing an N-terminal residue, facilitating the target protein’s ubiquitination and degradation [149, 150]. Another ubiquitin ligase, Nedd4-2, regulates the stability of cell surface membrane proteins. It belongs to the E3 ligase family and is expressed in Distal Convoluted Tubules (DCTs) and cortical collecting ducts (CCDs) [151]. Compared to Nedd4, Nedd4-2 exhibits a more potent inhibitory effect on TRPV5/6 due to the WW1 and WW2 domains in Nedd4-2 functioning as molecular switches, restricting the ubiquitination of the HECT domain to TRPV5 [151]. The decrease in TRPV5 in Xenopus oocytes leads to the downregulation of Ca^2+^ absorption and Na^+^ current mediated by TRPV5 and TRPV6, which can also be downregulated by Nedd4-2 and Nedd4 [151].

In summary, the protein modification processes discussed above involve the coordinated interaction of numerous enzymes with protein molecules. Several protein factors, directly or indirectly related to osteoporosis, exert their effects on TRPV5. For a summary of these factors, please refer to Table 2.

**Table 2:** Relationship between TRPV5 and OP at different bone tissue levels

	Different bone tissue levels	With OP	References
TRPV5	Bone Phenotype	In TRPV5^-/-^mice, bone resorption is disordered and the thickness of the femur is decreased The absence of TRPV5 results in accelerated bone aging and disturbance of bone resorption	[18, 117]
	Bone Tissue Cells	In TRPV5^-/-^mice, the number and size of osteoclast nuclei are significantly higher than those in WT mice; however, the absorptive capacity of osteoclasts is severely impaired TRPV5 plays a crucial role in calcium transport within the resorption cavity of osteoclasts E_2_ can enhance the expression of TRPV5 and stimulate osteoclast apoptosis	[18, 121, 123, 164]
	pH	External H^+^ influences the biological activity of TRPV5 by changing the conformation of TRPV5 “kiss and linger” way to affect TRPV5 activity	[73, 135]
	Molecular mechanism	Binds to CaR and CaM proteins to regulate Ca^2+^ concentration. Several post-translational modifications (PTMs) play a role in modulating the activity of TRPV5: • Phosphorylation-The cAMP-PKA pathway promotes the influx of Ca^2+^ • Glycosylation-vitamin D can upregulate Klotho and TRPV5, thereby regulating calcium concentrations in the blood • Ubiquitination- through the E 1 to 3, Nedd 4–2, TRPV5 protein function is adjusted	[11, 74, 143, 151]
	Activation and inhibition	PTH, VD_3_, CaR, Klotho, PI(4,5)P_2_, and others are common TRPV5 agonists that activate the channel’s activity and influence bone metabolism In osteoclasts, econazole inhibits the expression of TRPV5 in a dose-dependent manner, and it can suppress bone resorption by OCs in rats Oxoglaucine primarily obstructs the calmodulin transport pathway of TRPV 5 and suppresses Ca^2+^ influx, thereby inhibiting TRPV5 activity and promoting chondrocyte autophagy Plasmin can influence the binding of calmodulin to TRPV5 and reduce the activity of the TRPV5 channel	[121, 152, 162, 163]

### Activation and inhibition of TRPV5 channel

Nie et al. reported that TRPV5 is highly sensitive to calcium, and chemicals exhibiting specificity and potency may hold therapeutic significance for diseases related to imbalances in calcium homeostasis, such as hypercalcemia, renal calculi, and osteoporosis. TRPV5 can be activated by various agonists, including PTH, VD_3_, CaR, Klotho, PI(4,5)P_2_, and others, through different pathways, which can enhance its activity and function [152]. The discovery of agonists and inhibitors is critical to understanding their action sites and functions and can provide a reference for the development of drug targets and functionalities. Moreover, some drugs used to treat specific diseases can also stimulate or inhibit TRPV5.

#### Agonists of TRPV5

Regarding agonists, the β-adrenergic receptor (β-AR) comprises β1-AR, β2-AR, and β3-AR, with β1-AR and β2-AR primarily distributed in the renal DCT2/CNT [153]. The β1-AR agonist, dobutamine, upregulates the expression of cAMP in HEK293 cells, stimulates TRPV5 in Ca^2+^ uptake, and enhances the activity of the TRPV5 channel by phosphorylating T709 residues via the PKA pathway [154]. Streptozotocin-induced diabetes significantly increases TRPV5 mRNA expression in rats, and renal immunofluorescence sections demonstrate a significant increase in TRPV5 expression [155]. Diabetic patients exhibit increased calcium and magnesium levels in their urine, which is accompanied by an increase in the expression of TRPV5 and calcium-binding protein. Insulin treatment can reverse these trends [155]. Claudin-16 (CLDN16) plays a crucial role in Ca^2+^ and Mg^2+^ transport in the renal paracellular epithelium [156]. CLDN16-deficient mice display hypercalciuria and hypomagnesemia, similar to humans, as the ion compensation pathway emerges. The response of CLDN16-deficient mice is similar to that of humans. Mice exhibit hypercalciuria and hypomagnesemia [156]. Related hormones, such as PTH and 1,25(OH)_2_D_3_, and some Ca^2+^ and Mg^2+^ transport channels, including TRPV5, TRPM6, and calbindin-D9k, are significantly upregulated [156]. Glucocorticoids (GCs), such as dexamethasone (Dex) and dexmedetomidine, are widely used clinical anti-inflammatory and anti-allergic drugs that can cause side effects like osteoporosis due to abnormal bone metabolism [157]. Dex administration in mice induces the expression of TRPV5 transcripts in the kidney and TRPV6 transcripts in the duodenum within 24 h. GCs may regulate the transcription of TRPV5 and TRPV6 in an organ-specific and time-dependent manner [157].

#### Inhibitor of TRPV5

Econazole is a small molecule drug commonly used to treat skin antifungal infections and has been shown to inhibit TRPV5/6 in some studies [158, 159]. In osteoclasts, econazole inhibits the expression of TRPV5 in a dose-dependent manner, but has no effect on the activity of osteoclasts while inhibiting bone resorption in rats [121]. Freezing electron microscopy observations revealed that the binding conformation of TRPV5 and econazole results in the movement of S1-S4 and S4-S5 away from the hole axis, accompanied by the conformational change of the S6 helix, leading to the shrinkage of the lower gate and closure of the channel [159, 160]. Interestingly, the conformational changes induced by econazole do not significantly affect the outer pore region of the TRPV5 channel [159]. The concentration range of econazole, TH-1177, and other inhibitors is only in the micromolar range [161].

To achieve higher specificity, researchers continuously screen new inhibitors. ZINC9155420 and ZINC17988990 bind to TRPV5 in a nonconductive conformation along the ionic conduction pathway. The binding sites of these inhibitors do not overlap with activators, indicating that their effects do not result from competitive binding. Instead, these inhibitors lock the channels and prevent agonists from reaching the activated state [161]. Two new inhibitor binding sites have been identified in the TRPV5 structure. The inhibitory binding site of ZINC9155420 is located between lipid interfaces, a monomer S4-S5 linker, and the adjacent monomer S6 helix. The other inhibitory site mediated by ZINC17988990 occupies half of S1-S4 bundle cells, and its TRPV5 inhibitory specificity exceeds that of other reported compounds [161]. Oxoglaucine, a potential TRPV5 inhibitor, mainly blocks the calmodulin transport pathway of TRPV5 and inhibits Ca^2+^ influx, thereby inhibiting TRPV5 activity and activating chondrocyte autophagy [162]. In patients with nephrotic syndrome, calcareous deposition likely results from damage to the TRPV5 channel of DCT cells. Protease-activated receptor-1 (PAR-1) was purified from the urine of these patients by Kukiat Tudpor et al. [163]. PAR-1 can inhibit the uptake of Ca^2+^ by HEK293 cells. Plasmin-activated PAR-1-induced PKC-mediated phosphorylation of TRPV5 was found to affect the binding of calmodulin to TRPV5 and decrease the activity of the TRPV5 channel. It is speculated that urinary plasmin is an inhibitor of TRPV5 [163].

## Challenges and perspectives

Osteoporosis (OP) as a systemic metabolic disease is influenced by multiple factors and can be explored from histological, cytological, and molecular biology perspectives. In recent years, the impact of the microenvironment on OP has become a research hotspot. Dolores M. Shoback's team posits that new OP treatment strategies will focus on targeted drugs that eliminate senescent cells in the bone tissue microenvironment [165]. Moreover, a novel therapy for clearing senescent cells, called "senolytics," has been proposed, which can regulate bone loss caused by the high senescent cell state microenvironment [166]. This review examines the microenvironments at the bone tissue, cellular, and molecular levels in OP. Simultaneously, it introduces the essential calcium intake in the body, the channel switch TRPV5, and its relationship with OP from different levels, including bone tissue morphology, bone tissue cells, pH environment changes, molecular mechanisms, and the effects of agonists and inhibitors.

Research has shown that the absence of TRPV5 makes mice more susceptible to age-related osteoporosis [167]. Pumroy R.A. contends that the normal function of TRPV5/6 is closely related to OP and kidney calculi [168]. Recent studies on the pH microenvironment of bone tissue and TRPV5 reveal that the occurrence of metabolic acidosis reduces the expression and function of TRPV5 and the absorption of Ca^2+^ at least at the mRNA level [132, 169]. There are numerous types of hormones that can elicit changes in the level of TRPV5. For example, the relationship between serum vitamin D levels and TRPV5 is close, as demonstrated by the fact that a single injection of 1,25(OH)_2_D_3_ in vitamin D-deficient mice can increase the expression of TRPV5 mRNA in the kidney by 3–4 fold [170]. FGF23, derived from bone cells and acting on the kidneys, is a major factor in calcium and phosphorus regulation in the body. Osteoporosis patients typically have higher levels of FGF23. Klotho, combined with the FGF receptor, transforms into the specific receptor FGF23. α-K1, by binding to FGF23, can downregulate the production of vitamin D in the kidneys and activate the expression of TRPV5 [171, 172]. Therefore, TRPV5 has a complex relationship with various steroid hormones and small molecules in OP.

On the other hand, the process of TRPV5 exerting its protein function cannot be dissociated from various regulatory mechanisms, including transcription activation, intracellular transport, and post-translational modification [173]. Chen et al. discovered that administering soluble klotho to klotho-deficient mice can improve related phenotypes, extend lifespan, alleviate kidney fibrosis, and decelerate cellular aging, which is also beneficial in the glycosylation process of TRPV5 [174]. Moreover, the development of TRPV5 agonists and inhibitors as anti-osteoporosis drugs is the ultimate goal of related research. The development of TRPV5 inhibitors has rendered the channel a potential candidate for modulating the treatment of bone homeostasis. TRPV5/6 agonists can prevent postmenopausal osteoporosis (PMOP) induced by estrogen deficiency in postmenopausal women and optimize bone calcium supply [15, 175, 176].

In the future, the relationship between the osteoporosis microenvironment and TRPV5 will be more clearly elucidated, and drug development or disease diagnostic criteria centered on TRPV5 ion channels will provide more favorable conditions for the treatment of osteoporosis.

## Data Availability

Not applicable.
